# Pulse oximetry for children with pneumonia treated as outpatients in rural Malawi

**DOI:** 10.2471/BLT.16.173401

**Published:** 2016-10-11

**Authors:** Eric D McCollum, Carina King, Rashid Deula, Beatiwel Zadutsa, Limangeni Mankhambo, Bejoy Nambiar, Charles Makwenda, Gibson Masache, Norman Lufesi, Charles Mwansambo, Anthony Costello, Tim Colbourn

**Affiliations:** a Department of Pediatrics, Eudowood Division of Pediatric Respiratory Sciences, Johns Hopkins School of Medicine, Rubenstein Building, 200 North Wolfe Street, Baltimore, MD 21287, United States of America.; b Institute for Global Health, University College London, London, England.; c Parent and Child Health Initiative Trust, Lilongwe, Malawi.; d Ministry of Health, Lilongwe, Malawi.

## Abstract

**Objective:**

To investigate implementation of outpatient pulse oximetry among children with pneumonia, in Malawi.

**Methods:**

In 2011, 72 health-care providers at 18 rural health centres and 38 community health workers received training in the use of pulse oximetry to measure haemoglobin oxygen saturations. Data collected, between 1 January 2012 and 30 June 2014 by the trained individuals, on children aged 2–59 months with clinically diagnosed pneumonia were analysed.

**Findings:**

Of the 14 092 children included in the analysis, 13 266 (94.1%) were successfully checked by oximetry. Among the children with chest indrawing and/or danger signs, those with a measured oxygen saturation below  90% were more than twice as likely to have been referred as those with higher saturations (84.3% [385/457] vs 41.5% [871/2099]; *P* < 0.001). The availability of oximetry appeared to have increased the referral rate for severely hypoxaemic children without chest indrawing or danger signs from 0% to 27.2% (*P* < 0.001). In the absence of oximetry, if the relevant World Health Organization (WHO) guidelines published in 2014 had been applied, 390/568 (68.7%) severely hypoxaemic children at study health centres and 52/84 (61.9%) severely hypoxaemic children seen by community health workers would have been considered ineligible for referral.

**Conclusion:**

Implementation of pulse oximetry by our trainees substantially increased the referrals of Malawian children with severe hypoxaemic pneumonia. When data from oximetry were excluded, retrospective application of the guidelines published by WHO in 2014 failed to identify a considerable proportion of severely hypoxaemic children eligible only via oximetry.

## Résumé

**Objectif:**

Étudier l'utilisation de l'oxymétrie de pouls chez les enfants atteints de pneumonie en traitement ambulatoire au Malawi.

**Méthodes:**

En 2011, 72 prestataires de soins de santé de 18 centres de santé ruraux et 38 agents de santé communautaires ont été formés à l'utilisation de l'oxymétrie de pouls pour mesurer la saturation en oxygène de l'hémoglobine. Des données collectées entre le 1er janvier 2012 et le 30 juin 2014 par les individus formés sur des enfants âgés de 2 à 59 mois atteints d'une pneumonie cliniquement diagnostiquée ont été analysées.

**Résultats:**

Sur les 14 092 enfants inclus dans l'analyse, 13 266 (94,1%) ont été examinés avec succès grâce à l'oxymétrie. Parmi les enfants présentant un tirage sous-costal et/ou des signes de danger, ceux dont la saturation en oxygène mesurée était inférieure à 90% avaient plus de deux fois plus de chances d'être orientés vers un spécialiste que ceux présentant une saturation plus importante (84,3% [385/457] contre 41,5% [871/2099]; P < 0,001). L'utilisation de l'oxymétrie semble avoir augmenté de 0% à 27,2% (P < 0.001) le taux d'orientation vers un spécialiste dans le cas des enfants souffrant d'hypoxémie sévère sans tirage sous-costal ni signes de danger. En l'absence d'oxymétrie, si les directives en la matière publiées par l'Organisation mondiale de la Santé (OMS) en 2014 avaient été appliquées, 390/568 (68,7%) enfants souffrant d'hypoxémie sévère examinés dans les centres de santé considérés et 52/84 (61,9%) enfants souffrant d'hypoxémie sévère examinés par des agents de santé communautaires n'auraient pas pu bénéficier d'une orientation vers un spécialiste.

**Conclusion:**

L'utilisation de l'oxymétrie de pouls par les individus formés a considérablement accru l'orientation vers un spécialiste des enfants malawites atteints de pneumonie sévère et ayant développé une hypoxémie. Lorsque les données provenant de l'oxymétrie étaient exclues, l'application rétrospective des directives publiées par l'OMS en 2014 ne permettait pas d'identifier une proportion considérable d'enfants souffrant d'hypoxémie sévère qui remplissaient les conditions requises uniquement au vu de l'oxymétrie.

## Resumen

**Objetivo:**

Investigar la implementación de la oximetría de pulso ambulatoria en niños con neumonía en Malawi.

**Métodos:**

En 2011, 72 profesionales de la salud de 18 centros de salud en zonas rurales y 38 trabajadores de la salud comunitarios recibieron formación con respecto al uso de la oximetría de pulso para medir las saturaciones de oxígeno de la hemoglobina. Se analizaron los datos, que las personas formadas recopilaron entre el 1 de enero de 2012 y el 30 de junio de 2014, de niños de 2 a 59 meses de edad con un diagnóstico clínico de neumonía.

**Resultados:**

De los 14 092 niños incluidos en el análisis, se realizaron las pruebas con oximetría de manera satisfactoria a 13 266 (94,1%). Entre los niños con retracción costal y/o signos de peligro, aquellos con una medida de saturación de oxígeno por debajo del 90% tenían más del doble de probabilidades de haber sido derivados que aquellos con saturaciones más elevadas (84,3% [385/457] frente al 41,5% [871/2 099]; *P* < 0,001). La disponibilidad de la oximetría pareció haber incrementado la tasa de derivación para niños hipoxémicos graves sin retracción costal ni signos de peligro del 0% al 27,2% (*P* < 0,001). A falta de oximetría, si se hubieran aplicado las directrices de la Organización Mundial de la Salud (OMS) pertinentes publicadas en 2014, se podría haber considerado que 390/568 (el 68,7%) de los niños hipoxémicos graves en los centros sanitarios del estudio y 52/84 (el 61,9%) de los niños hipoxémicos graves visitados por los trabajadores de salud comunitarios no eran elegibles para la derivación.

**Conclusión:**

La implementación de la oximetría de pulso por las personas formadas incrementó de forma sustancial las derivaciones de los niños de Malawi con neumonía hipoxémica grave. Cuando se excluyeron los datos procedentes de la oximetría, la aplicación retrospectiva de las directrices publicadas por la OMS en 2014 no pudo identificar un porcentaje considerable de niños hipoxémicos graves que cumplían los requisitos únicamente a través de la oximetría.

## ملخص

**الغرض:**

الاستقصاء بشأن تنفيذ عمليات قياس التأكسج في العيادات الخارجية للأطفال المصابين بالالتهاب الرئوي في ملاوي.

**الطريقة:**

حصل 72 من مقدمي خدمات الرعاية الصحية في 18 مركزًا طبيًا ريفيًا و38 عاملاً في مجال الخدمات الصحية المجتمعية في عام 2011 على تدريبات متعلقة باستخدام تقنية قياس التأكسج بهدف قياس نسب تشبع الأكسجين والهيموجلوبين. وتم تحليل البيانات المجمعة في الفترة بين 1 كانون الثاني/يناير 2012 و 30 حزيران/يونيو 2014 بواسطة الأفراد المدرّبين، وهذه البيانات خاصة بالأطفال البالغ أعمارهم ما بين 2 إلى 59 شهرًا والمصابين بمرض الالتهاب الرئوي الذي تم تشخيصه داخل العيادات.

**النتائج:**

من بين 14092 طفلاً شملهم التحليل، كان 13266 طفلاً منهم (بنسبة ‏94.1‏%) قد تم فحصه بنجاح بواسطة تقنية قياس التأكسج. ومن بين الأطفال الذين يعانون من بروز القفص الصدري أثناء التنفس و/أو علامات الخطر، كان أولئك الذين لديهم نسبة تشبع الأكسجين أقل من 90‏% لديهم أرجحية مضاعفة لإحالتهم للعلاج باعتبارهم من الأطفال الذين لديهم نسبة تشبع أعلى (84.3‏% [385/457] مقابل ‏41.5‏% [871/2099[؛ *الاحتمال < 0.001 *). لقد بدا أن توافر تقنية قياس التأكسج قد زاد من معدل الإحالة للعلاج بالنسبة للأطفال المصابين بفشل الجهاز التنفسي الحاد من دون حدوث بروز للقفص الصدري أثناء التنفس أو ظهور علامات الخطر بنسبة تتراوح من 0‏% إلى 27.2‏% ( *الاحتمال * < 0.001). في حال تم تطبيق المبادئ التوجيهية النسبية لمنظمة الصحة العالمية والصادرة في عام 2014 في غياب تقنية قياس التأكسج، لكان 390/568 (68.7‏%) من الأطفال المصابين بخلل الجهاز التنفسي الحاد في مراكز الدراسة الصحية و 52/84 (‏61.9‏%) من الأطفال المصابين بخلل الجهاز التنفسي الحاد الذين تم اكتشاف إصابتهم من خلال العاملين في مجال الخدمات الصحية المجتمعية قد تم اعتبارهم غير مؤهلين للإحالة للعلاج.

**الاستنتاج:**

إن تنفيذ عملية قياس التأكسج بواسطة المتدربين لدينا أدى بشكل كبير إلى زيادة حالات الإحالة للعلاج بالنسبة للأطفال المصابين بالالتهاب الرئوي الحاد في ملاوي. وعندما تم استبعاد بيانات قياس التأكسج، فشل التطبيق بأثر رجعي للمبادئ التوجيهية الصادرة من منظمة الصحة العالمية في عام 2014 في تحديد نسبة كبيرة من الأطفال المصابين بخلل الجهاز التنفسي الحاد والذين كانوا مؤهلين للعلاج نتيجة استخدام تقنية قياس التأكسج فقط.

## 摘要

**目的:**

旨在调查马拉维肺炎患儿门诊脉搏血氧饱和度检测的实施情况。

**方法:**

2011 年，18 个农村医疗中心的 72 位医疗护理人员和 38 位社区医疗工作者接受了培训，学习使用脉搏血氧饱和度检测法测量血氧饱和度。 我们分析了受训人员于 2012 年 1 月 1 日到 2014 年 6 月 30 日期间采集的年龄在 2-59 个月、临床诊断为肺炎的儿童的数据。

**结果:**

在我们分析的 14 092 位儿童中，13 266 (94.1%) 位已经成功接受血氧饱和度检测。 在出现胸部凹陷和/或危险症状的儿童中，测量的血氧饱和度低于 90% 的儿童转诊的可能性是饱和度更高儿童的两倍以上（分别为 84.3% [385/457] 和 41.5% [871/2099]; *P* < 0.001）。 脉搏血氧饱和度检测的使用似乎将无胸部凹陷或危险症状的重度低氧血症儿童的转诊率从 0% 提高到了 27.2% (*P* < 0.001)。 在没有脉搏血氧饱和度检测的情况下，如果世界卫生组织 (WHO) 在 2014 年发布的相关指南已被采纳，则研究医疗中心的 390/568 (68.7%) 位重度低氧血症儿童和社区医疗工作者看诊的 52/84 (61.9%) 位重度低氧血症儿童会被认为不具备转诊资格。

**结论:**

受训人员对脉搏血氧饱和度检测的实施大幅度提高了马拉维重度低氧性肺炎患儿的转诊率。 但是如果不采用血氧饱和度检测中的数据，而是追溯式采纳 WHO 2014 年发布的指南，则相当大比例的仅通过血氧饱和度检测才能确诊的重度低氧血症儿童无法得到确诊。

## Резюме

**Цель:**

Изучить амбулаторное применение пульсоксиметрии у больных пневмонией детей в Малави.

**Методы:**

В течение 2011 года 72 врача из 18 сельских больниц и 38 местных медицинских работников были обучены использованию пульсоксиметрии для измерения насыщенности гемоглобина кислородом. Были проанализированы данные, собранные обученными лицами на протяжении периода с 1 января 2012 года по 30 июня 2014 года у детей в возрасте от 2 до 59 месяцев, которым был поставлен диагноз «пневмония».

**Результаты:**

Из 14 092 детей, включенных в анализ, у 13 266 (94,1%) удалось успешно провести пульсоксиметрию. Дети со впалой грудью и (или) опасными симптомами, у которых измеренная насыщенность кислородом была менее 90%, более чем в два раза чаще направлялись в больницу, чем дети с высокой оксигенацией (84,3% (385 из 457 детей) против 41,5% (871 ребенок из 2099), р < 0,001). Доступность оксиметрии привела к повышению уровня направления в больницу для детей с серьезной гипоксемией без впалой груди или опасных симптомов с 0 до 27,2% (p < 0,001). В отсутствие оксиметрии и при использовании соответствующих рекомендаций Всемирной организации здравоохранения (ВОЗ), опубликованных в 2014 году, 390 из 568 детей (68,7%) с серьезной гипоксемией в центрах проведения исследования и 52 из 84 детей (61,9%) с серьезной гипоксемией, которых осматривали местные медицинские работники, не были бы направлены в больницу ввиду отсутствия показаний.

**Вывод:**

Внедрение пульсоксиметрии нашими стажерами значительно повысило частоту направления в больницу малавийских детей с серьезной гипоксемией пневмонийного происхождения. При исключении из анамнеза данных пульсоксиметрии ретроспективное применение рекомендаций ВОЗ от 2014 года не позволяло выявить значительное количество детей с серьезной гипоксемией, необходимость госпитализации которых оказалось возможным определить только с помощью оксиметрии.

## Introduction

Among children with pneumonia, hypoxaemia is common, predicts mortality and is a marker of severe illness.^1^^–^^3^ Pulse oximetry – hereafter called oximetry – is the standard tool for non-invasively measuring peripheral arterial haemoglobin oxygen saturation. In low-income countries, however, access to oximetry has lagged behind access to life-saving oxygen treatment.^3^^–^^7^

In rural areas in low-income countries, there is interest in training community and health-centre-based health workers in oximetry – and making oximetry more widely available – so that more hypoxaemic children at risk of death can be referred to hospitals.^8^ Oximetry requires negligible infrastructure, is portable, non-invasive and user-friendly and offers a more accurate and objective way to identify hypoxaemia in children than clinical signs alone.^3^^,^^8^^,^^9^ Among practitioners and caregivers faced with decisions on the care of a child with severe pneumonia, the results of oximetry may be more persuasive than a clinical assessment alone.^10^

In late 2011, as part of a three-year prospective, observational study of the impact of a 13-valent pneumococcal conjugate vaccine, we introduced oximetry into two districts of central Malawi. One aim was to evaluate the usefulness of oximetry during the care, by rural health workers, of children aged 2–59 months with pneumonia.

## Methods

In our two study districts of Lilongwe and Mchinji, we assessed, prospectively, the quality of oxygen saturation measurement by oximetry and the impact of such measurement on referral decisions that were made according to the latest relevant Malawian guidelines. At the time of our study, the Malawian guidelines on pneumonia care at outpatient facilities and in the community^11^ were consistent with the World Health Organization’s (WHO’s) pre-2014 guidelines.^12^^,^^13^ We also estimated retrospectively the number of our hypoxaemic study children who would not have been referred if – as is usual for rural Malawi – oximetry had not been available and if the WHO 2014 guidelines on the integrated management of childhood illnesses – which do not recommend referral of pneumonia cases with chest indrawing at outpatient facilities^14^ – had been followed.

### Prospective routine care

In 2011, in our two study districts, we trained 110 health-care providers – i.e. 72 rural public-sector practitioners at 18 health centres and 38 community health workers (CHW) – in the use of oximetry, the keeping of medical records and other aspects of the care of children younger than five years with pneumonia. These providers had been selected, by the investigators and the Malawian Ministry of Health, because they worked in areas with consistent health services and were considered to be representative, in terms of paediatric health care, of rural Malawi. In these study areas, daily clinics in rural health centres were run by nurses or non-physician clinicians called clinical officers or medical assistants while salaried CHW – called health surveillance assistants – ran weekly village clinics. None of the study health centres provided oxygen.

The training lasted one day, cost approximately 13 United States dollars (US$) per trainee, was based on videos and small-group practical sessions and was run by a paediatric pulmonologist. Trainees were given lessons in the use, on children, of oximeters fitted with clip probes designed for adult fingers (Acare Technology, Xinzhuang, Taiwan, China). As supplied, by Lifebox Foundation (London, England), these oximeters and probes cost approximately US$ 250 and US$ 25 each, respectively. The trainees were advised to use a probe on a hallux – i.e. big toe – if the patient weighed less than 10 kg or was younger than two years and on an index finger of a heavier or older patient. An oximetric measurement was considered interpretable if it showed consistent, high-amplitude plethysmographic waveforms associated with a stable oxygen saturation. Providers were trained to diagnose children with pneumonia according to the Malawian 2000 guidelines supplemented with oximetry.^11^^–^^13^ Any findings of general danger signs, indrawing or severe hypoxaemia – i.e. an oxygen saturation of less than 90% – were to be considered indicative of the need for hospital referral. At the training’s conclusion, participants were evaluated and, if necessary, trained further. We retrained all of the trainees in early 2013.

After a three-month pilot study, all of the trained providers collected data on the children with clinically diagnosed pneumonia who they encountered between 1 January 2012 and 30 June 2014. The community-based and facility-based providers collected such data on routine care forms – as used for integrated community case management and the International Union Against Tuberculosis and Lung Disease’s Child Lung Health Programme, respectively – modified to include oximetry measurement results.^12^^,^^15^ The providers were not asked to perform any additional duties apart from the oximetry and they did not receive incentives. Members of the research team made monthly supervisory visits to each trained provider, at a monthly cost of about US$ 7 per trainee. Such visits provided opportunities to review the implementation of guidelines, appraise records, make direct observations of providers performing oximetry and interpreting the results and study the maintenance logs for the oximeters so that battery use, cleaning frequency and functionality could be determined. Any performance deficits observed were addressed.

### Mid-study quality assessment

In April 2013, we selected randomly – and separately assessed – the oximetric performance of 24 of our trained providers. We used 60 children without pneumonia – each awaiting elective surgery at Kamuzu Central Hospital in Lilongwe – as the test subjects. The results of the oximetric examination of the same subjects, by a paediatric pulmonologist (EDM), were used as the gold standard. Each provider was also asked to complete a multiple-choice and short-answer survey on their experience and problems with – and use of – oximetry.

### Missed hypoxaemia referrals

We investigated, retrospectively, the effects that applying different sets of guidelines with different oxygen saturation referral thresholds would have had on the referral to hospital of the paediatric pneumonia cases that our study providers encountered. With the data collected by the facility-based providers, we applied the Malawian 2000 guidelines – that recommend the referral of patients at health facilities because of indrawing – and the WHO 2014 guidelines – that do not recommend such referral. Our aim was to estimate the numbers of moderately hypoxaemic children – i.e. children with oxygen saturations of 90–92% – and severely hypoxaemic children, among all eligible children and also among all hypoxaemic children, who would not have been referred if oximetry had been unavailable. We conducted a similar analysis of the data from the CHW but, for pneumonia cases seen by such workers, both the Malawian and WHO guidelines that we considered recommend referral because of indrawing.

### Data analysis

For our analyses we used three sets of data: (i) the routine care forms completed by the trained providers; (ii) the maintenance logs for the oximeters; and (iii) the results of the mid-study quality assessment. Normally distributed data were described using means and standard deviations and compared in Student’s *t*-tests. Nonparametric data were described using medians and interquartile ranges and compared in Wilcoxon Mann–Whitney tests. Proportions were compared in Pearson *χ^2^* tests. For the quality assessment, the level of agreement between each provider and the expert (EDM) was expressed as a weighted kappa. To account for the accuracy of the oximeter used,^16^ oxygen saturation values that differed by no more than two percentage points were considered to be in full agreement. Agreements that gave weighted kappas of no more than 0.00 or of 0.01–0.19, 0.20–0.39, 0.40–0.59, 0.60–0.79 and 0.80–1.0 were categorized as poor, slight, fair, moderate, substantial and perfect, respectively.^17^ All analyses were performed using Stata version 13.1 (StataCorp. LP, College Station, USA).

### Ethical approval

The ethical boards of University College London (protocol 2006/002) and the Malawi Ministry of Health (protocol 941) approved the study protocol and did not require written consent, from the cases, for the collection of data on the routine care of pneumonia cases.

## Results

### Mid-study quality assessment

Twenty-two of the 24 randomly selected providers responded to the survey questions and were observed measuring oxygen saturation (Table 1 and Table 2). Nearly 94% (1222) of the 1301 successful measurements made by providers were within two percentage points of the expert’s measurements. The weighted kappa for the overall level of agreement between the providers and the expert (0.41; Table 2) indicated moderate agreement.

**Table 1:** Survey-based assessment of pulse oximetry use by health-care providers, Malawi, 2012–2014

Variable	All providers (*n* = 22)	HCB providers			CHW (*n* = 6)
		Medical assistants (*n* = 6)	Clinical officers (*n* = 5)	Nurses (*n* = 5)	
**Provider**					
Years in current job, mean (SD)^a^	5.8 (3.7)	4.3 (2.2)	4 (1.9)	11.6 (2.1)	3.8 (1.7)
Used pulse oximetry for > 12 months, no. (%; 95% CI)^a^	16 (72.7; 49.8–89.3)	4 (66.7; 22.3–95.7)	4 (80.0; 28.4–99.5)	4 (80.0; 28.4–99.5)	4 (66.7; 22.3–95.7)
**Daily number of measurements, mean (SD)**	18 (23)	7 (4)	21 (9)	40 (46)	7 (4)
**Mean measurement time, no. (%; 95% CI)**					
< 2 minutes	10 (45.5; 24.3–67.8)	5 (83.3; 35.9–99.6)	3 (60.0; 14.7–94.7)	2 (40.0; 5.3–85.3)	0 (0; 0–45.9)
2–5 minutes	12 (54.5; 32.2–75.6)	1 (16.7; 0.4–64.1)	2 (40.0; 5.3–85.3)	3 (60.0; 14.7–94.7)	6 (100.0; 54.1–100.0)
**Use of pulse oximetry, no. (%; 95% CI)**					
On children with cough or difficult breathing only	6 (27.3; 10.7–50.2)	3 (50.0; 11.8–88.2)	0 (0; 0–52.2)	1 (20.0; 0.5–71.6)	2 (33.3; 4.3–77.7)
On severely ill children, with or without cough or difficult breathing	12 (54.5; 32.2–75.6)	3 (50.0; 11.8–88.2)	4 (80.0; 28.4–99.5)	1 (20.0; 0.5–71.6)	4 (66.7; 22.3–95.7)
On other children	4 (18.2; 5.2–40.3)	0 (0; 0–45.9)	1 (20.0; 0.5–71.6)	3 (60.0; 14.7–94.7)	0 (0; 0–45.9)
**Challenges experienced, no. (%; 95% CI)**					
Battery charge difficult to maintain	15 (68.2; 45.1–86.1)	6 (100.0; 54.1–100.0)	4 (80.0; 28.4–99.5)	2 (40.0; 5.3–85.3)	3 (50.0; 11.8–88.2)
Clip probe not fitting well	14 (63.6; 40.7–82.8)	6 (100.0; 54.1–100.0)	3 (60.0; 14.7–94.7)	3 (60.0; 14.7–94.7)	2 (33.3; 4.3–77.7)
Child crying	5 (22.7; 7.8–45.4)	0 (0; 0–45.9)	2 (40.0; 5.3–85.3)	1 (20.0; 0.5–71.6)	2 (33.3; 4.3–77.7)
Child movement issues	5 (22.7; 7.8–45.4)	1 (16.7; 0.4–64.1)	2 (40.0; 5.3–85.3)	0 (0; 0–52.2)	2 (33.3; 4.3–77.7)
Child’s extremity too dirty for probe	19 (86.4; 65.1–97.1)	6 (100.0; 54.1–100.0)	5 (100.0; 47.8–100.0)	4 (80.0; 28.4–99.5)	4 (66.7; 22.3–95.7)

CHW: community health workers; CI: confidence interval; HCB: health-centre-based; SD: standard deviation.

^a^ At the mid-point of the study period, on 1 April 2013.

**Table 2:** Direct observational assessment of pulse oximetry use by health-care providers, Malawi, 2012–2014

Variable	All providers (*n* = 22)	HCB providers			CHW (*n* = 6)
		Medical assistants (*n* = 6)	Clinical officers (*n* = 5)	Nurses (*n* = 5)	
**SpO_2_ measurement quality, no. of measurements/total no. observed (%; 95% CI)**					
Use of hallux if patient weighed < 10 kg	733/790 (92.8; 90.8–94.5)	199/219 (90.9; 86.2–94.3)	162/179 (90.5; 85.2–94.4)	165/173 (95.4; 91.1–98.0)	207/219 (94.5; 90.6–97.1)
Patient calm when measurement made	1287/1320 (97.5; 96.5–98.3)	353/360 (98.1; 96.0–99.2)	292/300 (97.3; 94.8–98.8)	286/300 (95.3; 92.3–97.4)	356/360 (98.9; 97.2–99.7)
**Mean weighted kappa^a^**	0.41	0.51	0.40	0.36	0.37

CHW: community health workers; CI: confidence interval; HCB: health-centre-based; SpO_2_: peripheral arterial haemoglobin oxygen saturation.

^a^ For level of provider–expert agreement on measured oxygen saturations.

### Prospective routine care

During our study, we introduced 56 oximeters with probes. Thirteen (23.2%) oximeters and 24 (42.9%) probes were replaced during the study period.

**Fig. 1:**
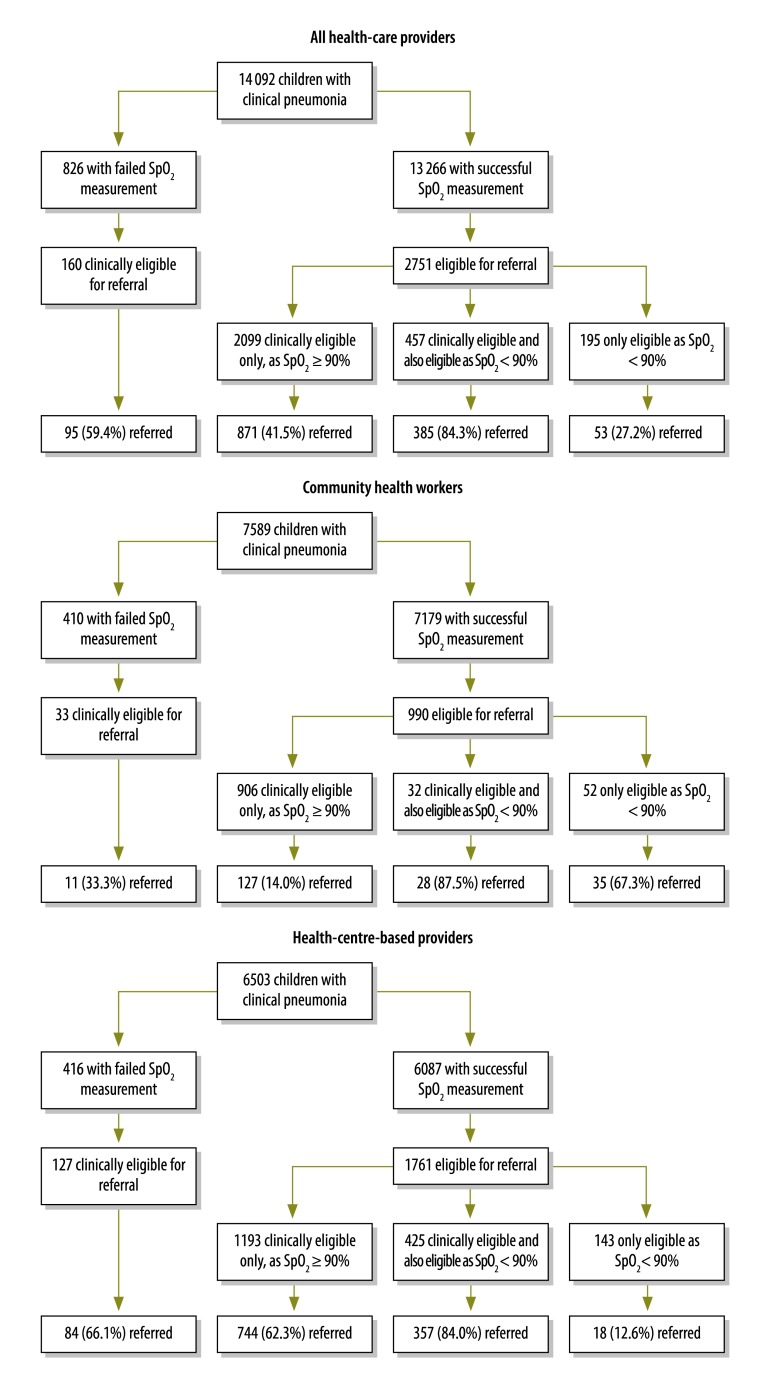
The pulse oximetric investigation and assessment for hospital referral of children with clinical pneumonia, by rural health-care providers, Malawi, 2012–2014

Over the study period, the providers reportedly measured the oxygen saturations of 13 266 (94.1%) of the 14 092 children with pneumonia who they reviewed (Table 3 and Fig. 1). Although severe hypoxaemia was indicated by a greater proportion of the oxygen saturations recorded by facility-based providers than by CHW [9.3% (568/6087) vs 1.2% (84/7179); *P* < 0.001], moderate hypoxaemia showed the opposite trend [8.9% (543/6087) vs 10.3% (627/7179); *P* = 0.007].

**Table 3:** Success and failure in the measurement of peripheral oxygen saturations of children aged 2–59 months with clinical pneumonia, Malawi, 2012–2014

Variable	Measurements by HCB providers					Measurements by CHW				All PPC seen by HCB providers (*n* = 6503)	All PPC seen by CHW (*n* = 7589)	*P*
	Successful (*n* = 6087)		Failed (*n* = 416)	*P*		Successful (*n* = 7179)	Failed (*n* = 410)	*P*				
**SpO_2_**												
Median value, % (IQR)	96.0 (94.0–98.0)	NA		NA		97.0 (95.0–98.0)	NA	NA		NA	NA	< 0.001
Patient with SpO_2_ of < 90%, no. (%)	568 (9.3)	NA		NA		84 (1.2)	NA	NA		NA	NA	< 0.001
Patient with SpO_2_ of 90–92%, no. (%)	543 (8.9)	NA		NA		627 (10.3)	NA	NA		NA	NA	0.007
**Age**												
Median value, months (IQR)	12.0 (7.0–23.0)	11.0 (6.0–19.0)		0.002		19.0 (9.0–34.0)	19.0 (10.0–33.0)	0.725		12.0 (7.0–22.0)	19.0 (9.0–34.0)	< 0.001
Patient aged 2–11 months, no. (%)	2805 (46.1)	220 (52.9)		0.007		2255 (31.4)	118 (28.8)	0.264		3025 (46.5)	2373 (31.3)	< 0.001
**Female, no. (%)**	2726/5701 (47.8)	146/314 (46.5)		0.649		3653/7101 (51.4)	196/407 (48.2)	0.209		2872/6015 (47.7)	3849/7508 (51.3)	< 0.001
**Weight in kg, mean (SD)^a^**	9.2 (2.8)	8.8 (2.6)		0.012		NR	NR	NR		9.1 (2.7)	NR	
**Respiratory rate in breaths/min, mean (SD)^b^**	53.4 (10.1)	55.1 (10.3)		0.048		50.4 (10.3)	49.8 (9.3)	0.264		53.9 (10.1)	50.3 (10.2)	< 0.001
**Chest indrawing, no. (%)**	1401 (23.0)	101 (24.3)		0.554		104 (1.4)	11 (2.7)	0.047		1502 (23.1)	115 (1.5)	< 0.001
**General danger signs, no. (%)^c^**	538 (8.8)	61 (14.7)		< 0.001		852 (11.9)	23 (5.6)	< 0.001		599 (9.2)	875 (11.5)	< 0.001
**Referral eligible, no. (%)**	1761 (28.9)	127 (30.5)		0.487		990 (13.8)	33 (8.0)	< 0.001		1888 (29.0)	1023 (13.5)	< 0.001

CHW: community health workers; HCB: health-centre-based; IQR, interquartile range; NA: not applicable; NR: not recorded; PPC: paediatric pneumonia cases; SD, standard deviation; SpO_2_: peripheral arterial haemoglobin oxygen saturation.

^a^ Weights were not recorded for 180 health-centre-based patients – i.e. 85 with, and 95 without, successful measurements of oxygen saturation – or, because community health workers did not have weighing scales, for any patients investigated in their communities.

^b.^ Respiration rates were not recorded for 264 health-centre-based patients – i.e. 140 with, and 124 without, successful measurements of oxygen saturation – or for 156 of the patients of community health workers - i.e. 130 with, and 26 without, successful measurements of oxygen saturation.

^c^ Patients were considered to have a general danger sign if they were abnormally sleepy, had convulsions, were not breastfeeding or drinking, were vomiting everything they ingested, showed stridor when calm, were infected with – or had been exposed to – human immunodeficiency virus and/or had severe malnutrition.

SpO_2_: peripheral arterial haemoglobin oxygen saturation.

Compared with the pneumonia cases described by the facility-based providers, those described by the CHW were, in general, older and had slower respiratory rates, higher median oxygen saturations and lower prevalences of indrawing and eligibility for referral according to the Malawian guidelines enhanced with oximetry (Table 3). Among the cases described by facility-based providers, those with failed oxygen saturation measurements were, in general, younger, weighed less and showed a higher prevalence of general danger signs than those with successful measurements (Table 3). Among the cases described by CHW, those with failed oxygen saturation measurements had lower prevalences of general danger signs and referral eligibility than those with successful measurements (Table 3).

Together, the trained providers were more than twice as likely to have referred a case who was clinically eligible for referral when the child had severe hypoxaemia than when the child did not (84.3% [385/457] vs 41.5% [871/2099]; *P* < 0.001; Fig. 1). If we assume that no clinically ineligible child with severe hypoxaemia would have been referred in the absence of oximetry, the availability of such oximetry appears to have increased referrals of such children by 27.2% – i.e. from zero to 27.2% (53/195; *P* < 0.001). The results of the trained providers’ responses to their oximetric measurements – i.e. in terms of referring pneumonia cases – are summarized in Fig. 1. Compared with the facility-based providers, the CHW correctly referred a greater proportion of severely hypoxaemic children who did not have indrawing or general danger signs (67.3% [35/52] vs 12.6% [18/143]; *P* < 0.001) and a lower proportion of children with either indrawing or danger signs and an oxygen saturation of at least 90% (14.0% [127/906] vs 62.3% [744/1193]; *P* < 0.001).

### Missed hypoxaemia referrals

**Fig. 2:**
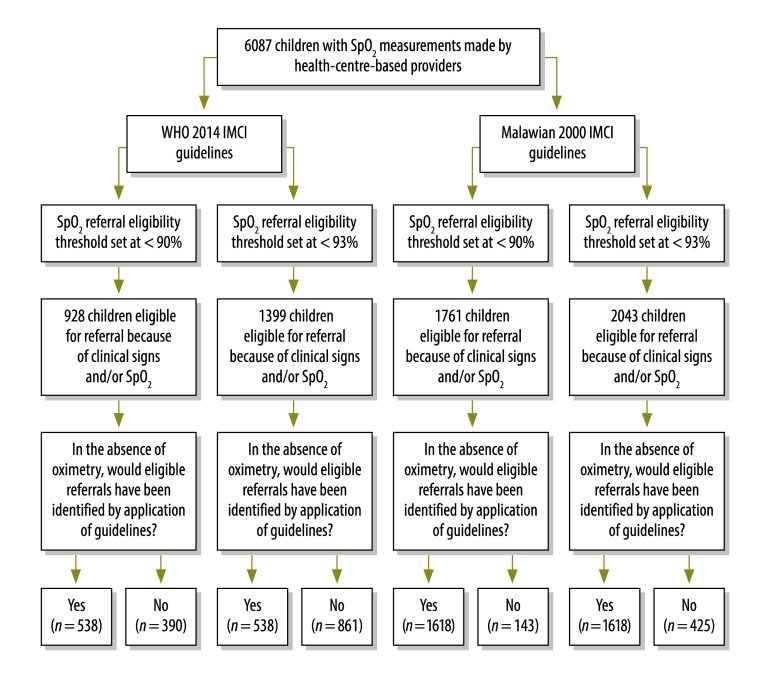
Estimated effects of the guidelines applied on hospital referrals among paediatric pneumonia cases investigated by health-centre-based health-care providers, Malawi, 2012–2014

IMCI: integrated management of childhood illnesses; SpO_2_: peripheral arterial haemoglobin oxygen saturation; WHO: World Health Organization.

Notes: Data collected by 72 providers were used to estimate the numbers of children with pneumonia who, though eligible for hospital referral on the basis of their clinical signs and/or peripheral arterial haemoglobin oxygen saturations, would not have been referred had either of two sets of relevant guidelines been applied without oximetry. In determining eligibility for referral, we considered two different oxygen saturation thresholds. The guidelines considered were those published, on the integrated management of childhood illnesses, by the World Health Organization in 2014^14^ or by the Malawian Ministry of Health in 2000.^11^ For facility-based health care providers, only the Malawian guidelines recommend the referral to hospital of paediatric pneumonia cases who show chest indrawing but no general danger signs.

We estimated the numbers of children found to have moderate or severe hypoxaemia – among all eligible children and among all children with hypoxaemia – who, in the absence of oximetry, would not have been referred if the providers had followed the relevant WHO guidelines published in 2014 or the latest Malawian guidelines and used either moderate or severe hypoxaemia as the referral threshold. If the facility-based providers had followed the WHO 2014 guidelines, 390 children with severe hypoxaemia – among 928 children eligible for referral (42.0%) using the severe hypoxaemia threshold (390/568 [68.7%] severely hypoxaemic children) – or 861 moderately or severely hypoxaemic children – among the 1399 children eligible for referral (61.5%) using the moderate hypoxaemia threshold (861/1111 [77.5%] moderately or severely hypoxaemic children) – would not have been referred (Fig. 2). If the same providers had followed the latest relevant Malawian guidelines, which do recommend referral because of chest indrawing alone, 143 children with severe hypoxaemia – among 1761 children eligible for referral (8.1%) using the severe hypoxaemia threshold (143/568 [25.2%] severely hypoxaemic children) – or 425 moderately or severely hypoxaemic children – among 2043 children eligible for referral (20.8%) using the moderate hypoxaemia threshold (425/1111 [38.3%] moderately or severely hypoxaemic children) – would not have been referred (Fig. 2).

**Fig. 3:**
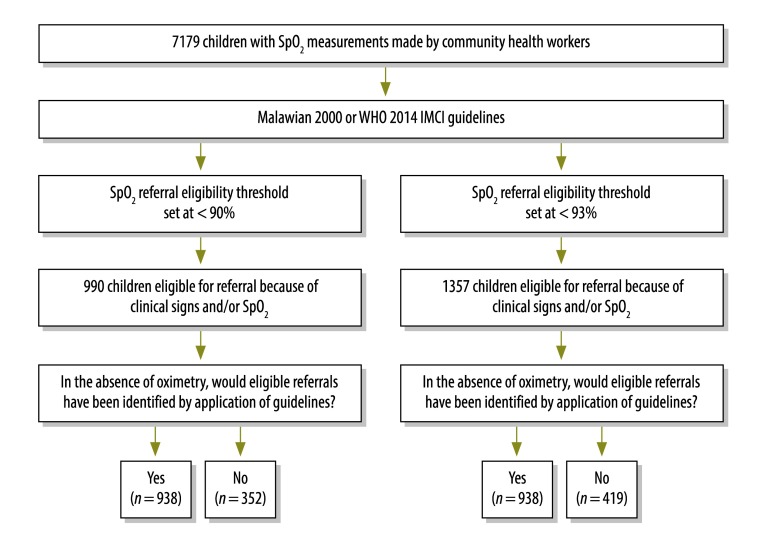
Estimated effects of the guidelines applied on hospital referrals among paediatric pneumonia cases investigated by community health workers, Malawi, 2012–2014

IMCI: integrated management of childhood illnesses; SpO_2_: peripheral arterial haemoglobin oxygen saturation; WHO: World Health Organization.

Notes: Data collected by 38 community health workers were used to estimate the numbers of children with pneumonia who, though eligible for hospital referral on the basis of their clinical signs and/or peripheral arterial haemoglobin oxygen saturations, would not have been referred had either of two sets of relevant guidelines been applied without oximetry. In determining eligibility for referral, we considered two different oxygen saturation thresholds. The guidelines considered were those published, on integrated community case management, by the World Health Organization in 2014^12^ or by the Malawian Ministry of Health in 2000.^11^ These two sets of guidelines are similar in terms of their recommendations for community health workers.

If the trained CHW had followed either the WHO 2014 or the Malawian 2000 guidelines, 52 severely hypoxaemic children – among 990 children eligible for referral (5.3%) when using the severe hypoxaemia threshold (52/84 [61.9%] severely hypoxaemic children) – or 419 moderately or severely hypoxaemic children – among 1357 children eligible for referral (30.9%) when using the moderate hypoxaemia threshold (419/711 [58.9%] moderately or severely hypoxaemic children) – would not have been referred in the absence of oximetry (Fig. 3).

## Discussion

This study examines the outpatient use of oximetry by front-line health workers at health centres and in the communities of a low-resource country. We analysed more than 15 400 oxygen saturation values, collected by health providers over three years and across two central Malawian districts, to assess the quality of the oximetric measurements, decision-making and – under existing clinical guidelines and the assumption that oximetry was unavailable – the failure to refer moderately or severely hypoxaemic cases. In general, we showed that the overall burden of hypoxaemia in our study area was high and that implementation of oximetry in Malawi, by front-line facility-based providers and CHW, would probably increase the referral of severely hypoxaemic children during routine pneumonia care. While we concluded that the quality of the oximetric measurements made by our trained providers was generally good, equipment durability and referral-related decision-making needs improvement. In the absence of oximetry, guidelines that do not recommend referral because of chest indrawing alone – e.g. the WHO 2014 guidelines on the integrated management of childhood illnesses – hamper the referral of substantial proportions of moderately or severely hypoxaemic Malawian children who may be at high risk of early death.

Our results show that, while not without challenges, rural health workers can use oximetry with moderate reliability. Although the overall weighted kappa that we calculated for the level of provider–expert agreement (0.41) compares favourably with the kappa reported, for inter-observer agreement on child chest indrawing, in the United Republic of Tanzania (0.33),^18^ it was lower than we expected. The probable reasons for the relatively low weighted kappa we calculated include the natural temporal variation in one child’s oxygen saturation, the influences of movement artefact and poor perfusion on measurement accuracy and the inherent accuracy of the oximeters that were used.

We found the failure of health-care providers using an oximeter to measure oxygen saturation to be associated with certain patient characteristics. In health centres, for example, failure tended to occur with patients who were sicker, younger and smaller than most of the other pneumonia cases that our trained providers encountered. We suggest that a failed measurement in a health centre could be a referral indication. Poor extremity perfusion in a dehydrated or septic child increases the likelihood of measurement failure, especially if the probe is not ideally sized.^6^ Our trained CHW appeared to struggle most with recording the oxygen saturation of mildly ill toddlers. As such children are often active, agitated and anxious with strangers, they may represent a particular challenge to providers who are trying to minimize movement artefact as they measure a child’s oxygen saturation.^6^ While we considered using paediatric clip or wrap probes in our study, they were more expensive than adult finger clips at the time of our study. One of our aims was to investigate the implementation of equipment that might be affordable when used on a large scale. In low-income countries, low-cost, durable paediatric probes that fit a range of patient sizes while providing precise measurements in less than 30 seconds are likely to be an important factor in the successful implementation of oximetry at a national level. The oximeters we investigated were designed for use in the operating theatres of low-resource countries – not in rural communities. Lifebox Foundation is now designing a low-cost oximeter for use, by front-line health workers, on children (I Walker and I Wilson, personal communication, 2016).

Although the referral-related decision-making we investigated sometimes appeared inconsistent, we expected this since, in Malawi, the referral of a child is typically a joint decision, between the provider and caregiver, that is influenced by many social factors – e.g. the childcare available and domestic responsibilities and finances. Our results indicate that, when making referral decisions, providers might perform substantially better when clinically eligible children are also identified as severely hypoxaemic. The data we analysed do not provide contextual, qualitative information as to why some severely hypoxaemic children were referred when others were not. In the future, we plan to conduct focus group discussions to understand the providers’ decision-making better. Despite the providers’ mixed performance in making referral decisions, their use of oximetry led to the referral of about 248 [385-(41.5%*457)+53=248] severely hypoxaemic children who would probably not have been referred in the absence of oximetry (Fig. 1).

When oximetry is not available – i.e. the normal situation in rural Malawi – providers who follow the Malawian 2000 guidelines or, to a greater extent, the WHO 2014 guidelines are likely to under-refer children with severe hypoxaemia. Among hospitalized children with pneumonia^3^^,^^9^ and, it seems, also among outpatients with pneumonia in Malawi, oximetry offers a more reliable method of identifying hypoxaemia than the observation of clinical signs. While research in developing countries has focused on severe hypoxaemia’s strong association with poor outcomes,^1^ recent evidence from a systematic review^19^ and from a study of hospitalized Malawian children^20^ indicates that even moderate hypoxaemia is also a predictor of death in childhood pneumonia. Given this evidence – and the fact that many moderately hypoxaemic children progress to severe hypoxaemia^21^ – all outpatients with pneumonia who have oxygen saturations below 93% should perhaps be referred. We need more outcome-based community-level research on this topic.

Our study had two main limitations. First, the data we analysed were largely collected during routine care and, although such follow-up is recommended, few Malawian children are re-evaluated after they have completed pneumonia treatment. We were therefore unable to determine patient outcomes and were even unable to confirm whether or not referred patients went to hospital. Second, since the study was observational and lacked a control group, our results should be interpreted with caution.

With refinement of the equipment and wider implementation, oximetry has potential to improve the outcomes of children with pneumonia and hypoxaemia in rural Malawi and other comparable settings. Without oximetry, the implementation of the WHO 2014 guidelines at Malawian outpatient facilities could result in high numbers of severely hypoxaemic children not receiving life-saving oxygen. While similar research in other settings with high pneumonia burdens is also needed – including outcome-based work that includes a control group and/or referral for moderate hypoxaemia – we believe this study supports the inclusion of oximetry in the WHO guidelines and the next set of relevant Malawian guidelines.
