# Corrigendum

**DOI:** 10.2471/BLT.17.100117

**Published:** 2017-01-01

In Volume 94, Issue 12, December 2016, page 899, Fig. 3, (*n* = 352) should have read (*n* = 52): McCollum ED, King C, Deula R, Zadutsa B, Mankhambo L, Nambiar B, et al. Pulse oximetry for children with pneumonia treated as outpatients in rural Malawi. Bull World Health Organ. 2016;94(12):899. http://dx.doi.org/10.2471/BLT.16.173401

